# Effects of sampling method on foliar *δ*^13^C of *Leymus chinensis* at different scales

**DOI:** 10.1002/ece3.1401

**Published:** 2015-02-09

**Authors:** Yanjie Liu, Yan Li, Lirong Zhang, Xingliang Xu, Haishan Niu

**Affiliations:** 1 College of Resources and Environment, University of Chinese Academy of Sciences19-A Yuquan Road, Beijing 100049, China; 2 Ecology, Department of Biology, University of Konstanz, Universitätsstrasse 10D-78457 Konstanz, Germany; 3 Key Laboratory and Ecosystem Network Observation and Modelling, Institute of Geographic Sciences and Natural Resources Research, Chinese Academy of SciencesNO.11-A Datun Road, Beijing, 100101, China

**Keywords:** Environmental variability, field experiment, sampling error, temperate steppes, transect

## Abstract

Stable carbon isotope composition (*δ*^13^C) usually shows a negative relationship with precipitation at a large scale. We hypothesized that sampling method affects foliar *δ*^13^C and its response pattern to precipitation. We selected 11 sites along a precipitation gradient in Inner Mongolia and collected leaves of *Leymus chinensis* with five or six replications repeatedly in each site from 2009 to 2011. Additionally, we collected leaves of *L. chinensis* separately from two types of grassland (grazed and fenced) in 2011. Foliar *δ*^13^C values of all samples were measured. We compared the patterns that foliar *δ*^13^C to precipitation among different years or different sample sizes, the differences of foliar *δ*^13^C between grazed and fenced grassland. Whether actual annual precipitation (AAP) or mean annual precipitation (MAP), it was strongly correlated with foliar *δ*^13^C every year. Significant difference was found between the slopes of foliar *δ*^13^C to AAP and MAP every year, among the slopes of foliar *δ*^13^C to AAP from 2009 to 2011. The more samples used at each site the lower and convergent *P*-values of the linear regression test between foliar *δ*^13^C and precipitation. Furthermore, there was significant lower foliar *δ*^13^C value in presence of grazed type than fenced type grassland. These findings provide evidence that there is significant effect of sampling method to foliar *δ*^13^C and its response pattern to precipitation of *L. chinensis*. Our results have valuable implications in methodology for future field sampling studies.

## Introduction

Stable carbon isotope composition (*δ*^13^C) of the leaves of C_3_ plants is largely related to the temporally averaged ratio of the concentration of intercellular to atmospheric CO_2,_ *c*_*i*_*/c*_*a*_, which is the result of the balance between stomatal conductance and photosynthesis (Farquhar et al. 1982, 1989). Factors that affect either stomatal conductance or photosynthesis also have effects on foliar *δ*^13^C. A close relationship exists between *c*_*i*_*/c*_*a*_ and plant water use efficiency (WUE), which means that foliar *δ*^13^C can provide an estimate of the integrated long-term WUE of a plant (Ehleringer and Cooper 1988; Farquhar et al. 1989; Bert et al. 1997; Silim et al. 2001; Michelot et al. 2011). Foliar *δ*^13^C is thus a complex trait involved in acclimation, adaptive processes.

Foliar *δ*^13^C values of C_3_ plants are known to be affected by environmental factors, with water availability in particular showing a strong negative relationship with foliar *δ*^13^C (Stewart et al. 1995; Swap et al. 2004; Liu et al. 2005; Liu, Tian et al. 2013; Liu, Xu et al. 2013; Liu et al. 2014). Many studies have reported a trend higher foliar *δ*^13^C values in drier environment (Hausmann et al. 2005; Schulze et al. 2006; Luo et al. 2009; Diefendorf et al. 2010; Prentice et al. 2011; Wang et al. 2013). Many transect studies also showed that foliar *δ*^13^C decreases with increasing precipitation (Swap et al. 2004; Guo and Xie 2006; Zheng and Shangguan 2006; Song et al. 2008; Prentice et al. 2011; Liu et al. 2013,b^2013^). However, very few studies have considered the effect of sampling method on foliar *δ*^13^C and its response pattern to precipitation.

**Figure 1:**
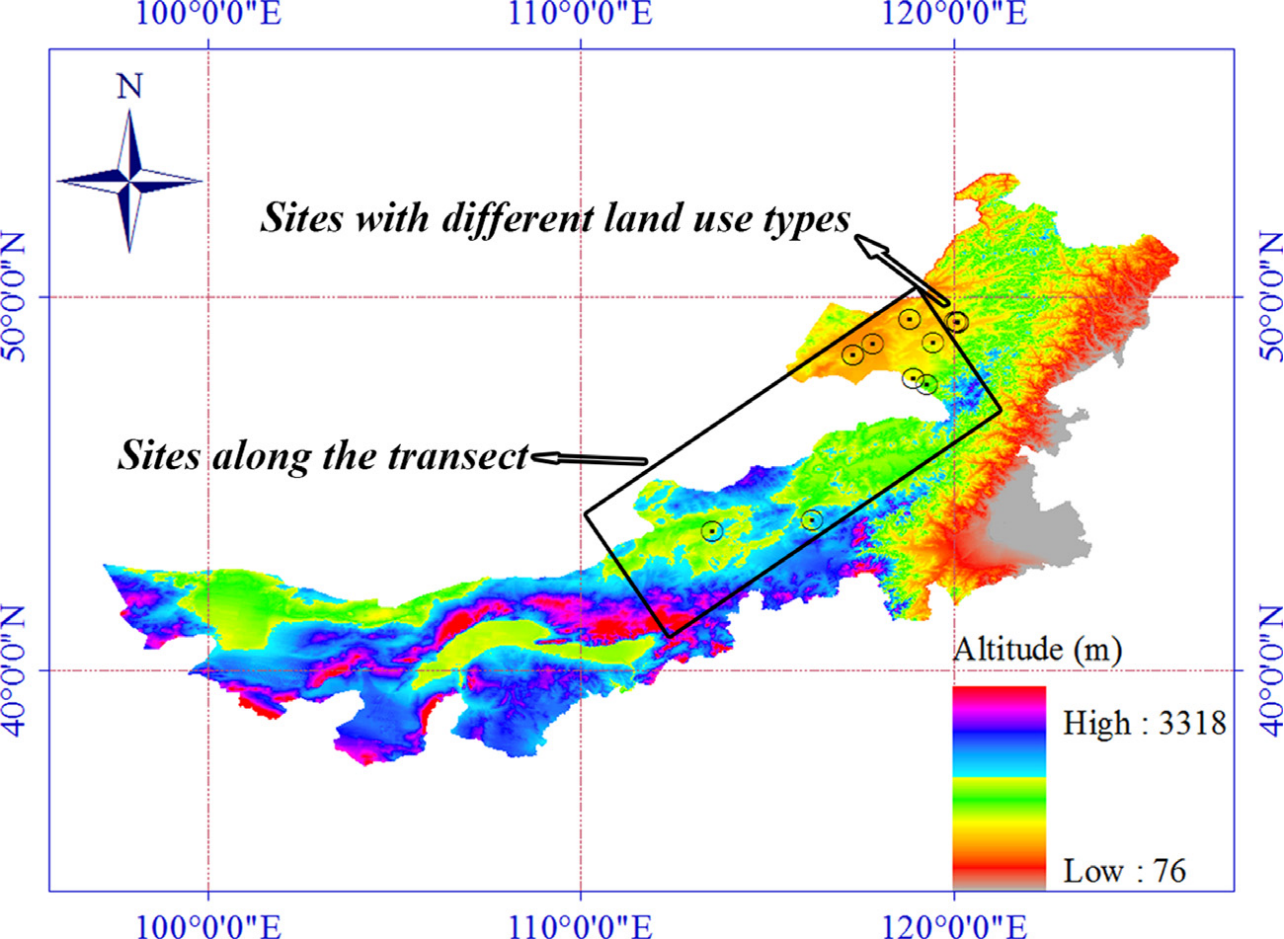
Location of sampling sites on the topographic map of Inner Mongolia, China.

Water availability is an important factor that influences plant growth (McConnaughay and Coleman 1999; Poorter and Nagel 2000), and variation in water availability drives variation in vegetation types. This is particularly pronounced in the temperate steppes of Inner Mongolia, China (Gong et al. 2011; Li et al. 2011; Liu et al. 2013,b^2013^). The central Eastern regions in the Inner Mongolia with undulating terrains are mainly divided into medium and low mountainous regions, and hills and peneplain regions (Fig.1). There is large spatial heterogeneity of water availability also among nearly microhabitats of this area, although they are exposed to similar climatic conditions. Some researchers found different foliar *δ*^13^C response patterns to precipitation for the same species (Su et al. 2000; Prentice et al. 2011), and the reason may be that they only collected one sample per site along a precipitation gradient and did not consider the heterogeneity of water availability among microhabitats.

Most of the grasslands are grazed by cattle in this region (Yan et al. 2013), and it is virtually impossible to find undisturbed grassland. However, it is also very common in Inner Mongolia that grassland is fenced in order to prevent its degradation from overgrazing. Few studies considered the impact of land-use types on foliar *δ*^13^C when they collected the samples at a large geographical scale. Furthermore, some studies used the mean annual precipitation (MAP) instead of actual annual precipitation (AAP) to test the relationship between foliar *δ*^13^C and precipitation at a large scale (Wittmer et al. 2008; Prentice et al. 2011). However, temporal variability of precipitation among years may also affect this relationship.

Herein, we set out to test the hypothesis that sampling design affects foliar *δ*^13^C and its response pattern to precipitation, by analyzing leaf samples of the same species distributed along a transect in the central Eastern regions of Inner Mongolia, China. Specifically, the following three questions are addressed: (1) Do the patterns of foliar *δ*^13^C values in response to precipitation differ among different years? (2) Do the numbers of samples per site affect the foliar *δ*^13^C response pattern to precipitation at a large scale? (3) Does different land use of grassland (grazed and ungrazed (fenced) grasslands) affect foliar *δ*^13^C values?

## Materials and Methods

### Study species

*Leymus chinensis* (Trin.) Tzvel. (Poaceae), a perennial rhizomatous C_3_ grass, shows both vegetative reproduction and sexual reproduction (The Integrated Investigation Team in Inner Mongolia and Ningxia, CAS 1985). It occupies large areas as a dominant or codominant species in the eastern parts of the Eurasian steppes and thrives in a diverse range of habitats (The Integrated Investigation Team in Inner Mongolia and Ningxia, CAS 1985; Liu et al. 2007). There are two ecotypes of this species that differ in leaf color: one is yellow-green and the other one is gray-green (Fig. S1). The yellow-green ecotype is only found in meadow steppes, while the gray-green one is distributed across a more extensive range of environmental conditions (Chen and Wang 2009). Furthermore, these two ecotypes also sometimes co-occur in the same region. We only assessed the foliar *δ*^13^C values of *L. chinensis* with gray-green leaf color in our study, because the yellow-green type only occurs in a small region of Inner Mongolia, and because the two ecotypes differ in foliar *δ*^13^C (Fig. S2), and we want to unravel the effects of sampling method on foliar *δ*^13^C at a large geographical scale.

### Large-scale transect study

We selected 11 sites along a west–east precipitation gradient in Inner Mongolia (Table1 and Fig.1). We collected samples from each site in late June 2009, and late August 2010 and 2011. Foliar *δ*^13^C values of C_3_ plants are known to be affected by water availability (Stewart et al. 1995; Liu et al. 2014). We avoided sites located near rivers, because water availability there is not only determined by precipitation. There is spatial heterogeneity (i.e., among microhabitats) within each site mostly due to topography, and therefore, we expected heterogeneity in foliar *δ*^13^C values. In order to encompass such heterogeneity and reduce the sampling error by avoiding sampling one specific microhabitat by chance, we first assessed the topography and identified a baseline along the hillside aspect at each site. Then, we systematically collected six samples (five in 2011) from six plots along the baseline. The distance between two adjacent plots along the baseline was 10 m. Foliar *δ*^13^C values can vary among leaves within a plant (Yang et al. 2011). Therefore, for each sample, we collected all mature leaves of 5–8 randomly chosen *L. chinensis* individuals and mixed them. Leaves were microwaved immediately (500 W, 2 min) after collection to make sure that plant enzymes were deactivated and then air-dried. Once back in the laboratory, the samples were further dried in a drying oven at 65°C for 48 h.

**Table 1:** Location and characteristics of the sites sampled along the precipitation gradient in Inner Mongolia, China, from 2009 to 2011

Site	Latitude (N)	Longitude (E)	Altitude (m a.s.l.)	AAP (mm)			MAP (mm)
				2009	2010	2011	
T1	43°43.21′	113°31.64′	1027	181.85	183.54	163.17	265.77
T2	44°01.31′	116°12.43′	1051	227.53	287.85	245.86	335.80
T3	48°27.20′	117°18.80′	624	314.47	182.16	258.22	330.89
T4	48°27.20′	117°18.80′	624	314.47	182.16	258.22	330.89
T5	48°46.46′	117°49.67′	550	329.64	201.92	275.01	348.28
T6	49°25.96′	118°48.21′	616	394.49	260.71	333.20	381.97
T7	49°25.96′	118°48.21′	616	394.49	260.71	333.20	381.97
T8	47°50.54′	118°54.99′	757	361.79	295.97	348.90	387.33
T9	47°50.54′	118°54.99′	757	361.79	295.97	348.90	387.33
T10	47°39.52′	119°17.44′	871	371.58	312.46	367.57	387.01
T11	48°46.79′	119°27.83′	680	407.34	306.82	373.12	397.19

APP, actual annual precipitation; MAP, mean annual precipitation.

For this study, we used climatic data recorded by 54 weather stations distributed along our transect. Based on these data, we used Kriging interpolation to determine the actual annual precipitation (AAP) at each sample location for 2009, 2010, and 2011. The mean annual precipitation (MAP) across the last 15 years was also calculated. Kriging interpolation was implemented in ArcGIS 10.0.

### Small-scale regional study

Grazed and fenced grasslands are two common land-use types in Inner Mongolia. In order to investigate how these two land-use types influence foliar *δ*^13^C, we conducted a small-scale regional study in early September 2011. We selected three sites in the Hulunbuir meadow steppe of Inner Mongolia, China (Table2 and Fig.1). Plant communities in these three sites are dominated by *L. chinensis*,*Stipa baicalensis*, and mesophytic forbs. Each site had both land-use types; one part was grazed by cattle animals, and another part was enclosed by a fence (Fig. S3). All fenced grasslands had been enclosed since 2006. For each land-use type at the three sites, we randomly placed three quadrats (1 m × 1 m), in which we collected all mature leaves of *L. chinensis* and mixed them as one sample. All samples were dried in an oven at 65°C for 48 h.

**Table 2:** Location and characteristics of the sites sampled in Hulunbuir meadow steppe of Inner Mongolia, China, in 2011

Site	Latitude (N)	Longitude (E)	Altitude (m a.s.l.)	Land-use type		Dominant species
1	49°22.33′	120°1.73′	633	Grazing	May to September	*Leymus chinensis*
				Fenced	Since 2006	*Leymus chinensis*
2	49°21.17′	120°6.15′	658	Grazing	May to September	*Stipa baicalensis*
				Fenced	Since 2006	*Stipa baicalensis*
3	49°19.33′	120°5.87′	619	Grazing	May to September	Mesophytic forbs
				Fenced	Since 2006	Mesophytic forbs

### Carbon isotope measurement

All collected plant material was ground to a homogenous powder using a ball mill (MM200; Retsch, Haan, Germany). Aliquots of approximately 2.5 mg of plant material were weighed into tin capsules for foliar *δ*^13^C measurement. For samples collected in 2009 and 2010, foliar *δ*^13^C was measured using continuous-flow gas isotope ratio mass spectrometry (CF-IRMS) with Vario PYRO Cube (IsoPrime100; Isoprime Ltd., Stockport, U.K.) at the Institute of Environment and Sustainable Development in Agriculture, CAAS, China. Vienna Pee Dee Belemnite (VPDB) was used as the reference standard for C isotopic analyses. Reproducibility was high as the standard deviation of repeated measurements was lower than 0.20‰. For samples collected in 2011, foliar *δ*^13^C was measured using CF-IRMS with Flash EA1112 and the interface Conflo III (MAT 253; Finnigan MAT, Bremen, Germany) at the Institute of Geographic Sciences and Natural Resources Research, Chinese Academy of Sciences, China. Pee Dee Belemnite (PDB) was used as the reference standard for C isotopic analyses. Reproducibility was high as the standard deviation of repeated measurements was lower than 0.15‰.

### Statistical analyses

All statistical analyses were performed using R 3.0.2 (R Core Team 2013). We tested for relationships between foliar *δ*^13^C and AAP in the study year, or MAP across the last 15 years, using linear regression. The slope of the regression line of foliar *δ*^13^C against AAP or MAP was the response strength of foliar *δ*^13^C, that is, indicating how strongly foliar *δ*^13^C responded to precipitation. In order to test whether foliar *δ*^13^C response to AAP differs from its response to MAP, the difference in response strength with respect to AAP and MAP was tested using the Standardized Major Axis Tests & Routines (SMATR) R package (Falster et al. 2006). In order to test whether foliar *δ*^13^C response to AAP varies among years, the difference in response strength with respect to AAP among years was also tested using SMATR. There were six samples in 2009 and 2010, and five samples in 2011 at each site along the transect. In order to test how strongly foliar *δ*^13^C responded to AAP at a large geographical scale when different sample sizes were used, we randomly sampled one, two, three, four, and five samples from each site, respectively. Therefore, we obtained different datasets, as each site along the transect had different samples. For these datasets, the relationship between foliar *δ*^13^C ratio and AAP was tested using linear regression. Furthermore, for the small-scale region study, in order to test the effects of sampling site and land-use type on foliar *δ*^13^C values, we did a two-way analysis of variance (ANOVA).

## Results

### Large-scale patterns of foliar *δ*^13^C in response to precipitation across years

**Figure 2:**
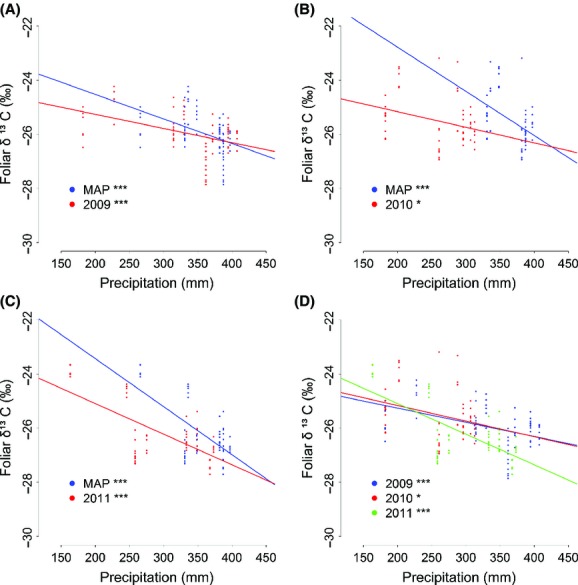
Variation in the response of foliar *δ*^13^C to precipitation. Response of foliar *δ*^13^C to mean annual precipitation (MAP, blue) and actual annual precipitation (AAP, red) in 2009 (A), 2010 (B), and 2011 (C). Response of foliar *δ*^13^C to AAP in 2009, 2010, and 2011 (D). Level of significance: “***”*P *<* *0.001, “**”*P *<* *0.01, “*”*P *<* *0.05.

Foliar *δ*^13^C values along the transect significantly decreased with increasing precipitation (whether AAP or MAP) in each year from 2009 to 2011 (Fig.2). However, the response of foliar *δ*^13^C to MAP was significantly stronger than the response to AAP (2009, *P *<* *0.001, Fig.2A; 2010, *P *<* *0.001, Fig.2B; 2011, *P *<* *0.001, Fig.2C). Furthermore, the response of foliar *δ*^13^C to AAP also significantly differed among the 3 years of sampling (*P *<* *0.001, Fig.2D).

**Figure 3:**
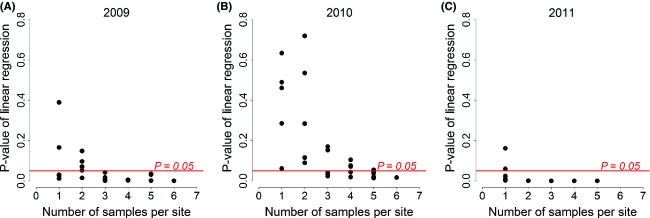
Effect of sample size (number of samples per site) on the *P*-values of the linear regression between foliar *δ*^13^C and precipitation. Samples collected in 2009 (A), 2010 (B), and 2011 (C).

Foliar *δ*^13^C values significantly decreased with increasing precipitation (AAP) for *L. chinensis* in any year from 2009 to 2011 (2009: *R*^2 ^= 0.201, *P *<* *0.001; 2010: *R*^2 ^= 0.095, *P *<* *0.05; 2011: *R*^2 ^= 0.449, *P *<* *0.001; Fig.2D and Table S1), when all samples at each site along the transect were analyzed. However, we found that with increasing sample size from the same site, *P*-values of the linear regression test for the slope if foliar *δ*^13^C against AAP decreased (Fig.3 and Table S1). Overall, five samples would be needed as a minimum in order to detest a significant relationship between precipitation and *δ*^13^C in this study.

### Influence of land-use type on foliar *δ*^13^C

**Figure 4:**
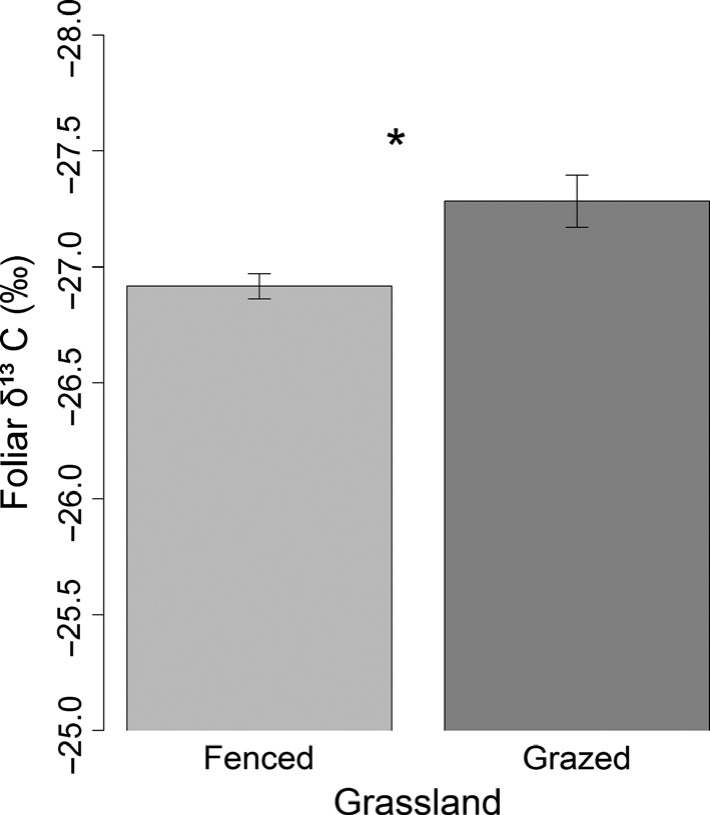
Difference in foliar *δ*^13^C under two different land-use types (fenced and grazing). Error bars represent standard errors of the means. Level of significance: “*”*P *<* *0.05.

Foliar *δ*^13^C significantly differed between grazed and fenced areas (*P *<* *0.05), with lower values in area with grazing (−27.28 ‰ ± 0.112‰) than in areas without grazing (−26.92‰ ± 0.055‰; Fig.4).

## Discussion

This study represents the comprehensive examination of the effect of sampling methods on foliar *δ*^13^C values. We focused on the effect of different sampling methods in a typical microhabitat and avoided obvious factors affecting foliar *δ*^13^C values, such as the sites near a river, the different ecotypes of *L. chinensis* (Figs. S1 and S2), and the leaf position (Yang et al. 2011). Our findings, obtained under optimum field sampling conditions in some microhabitats (50–60 m) along a precipitation gradient, provide evidence that there is significant effect of sampling methods on foliar *δ*^13^C: (1) the foliar *δ*^13^C response patterns were significantly different between to MAP and to AAP, whereas the response of foliar *δ*^13^C to AAP was also significantly different among 3 years from 2009 to 2011; (2) sample size from microhabitats strongly affects the patterns of foliar *δ*^13^C in relation to precipitation at a large geographical scale; (3) sampling from same sites with different land-use types (grazed and fenced) affects foliar *δ*^13^C values significantly.

Our results showed the common tendency that foliar *δ*^13^C values decreases with increasing precipitation (whether AAP or MAP) (Swap et al. 2004; Song et al. 2008; Prentice et al. 2011); however, the response sensitivity of foliar *δ*^13^C values to precipitation was significantly different when we used MAP instead of AAP to analyze the data (Fig.2A–C). The response sensitivity also differed even when only foliar *δ*^13^C to AAP among different years was compared (Fig.2D). The most likely explanation for these differences over time is that species growing among different habitats in the temperate steppes with significantly different response sensitivity of foliar *δ*^13^C values to precipitation (Liu et al. 2014), and inter-annual variation in precipitation makes foliar *δ*^13^C not on same level of fluctuation among different sites. Therefore, temporal variability of foliar *δ*^13^C in a microhabitat can affect its response patterns to precipitation at a large scale. Wittmer et al. (2008) reported that Δ^13^C among *Stipa* in Central Asian grassland increased with MAP in both 2 years and that the slope of this relationship (Δ^13^C to MAP) was very similar between 2 years. Contrast to our results of this study, it will be interesting to see how their results hold up when they test the homogeneity between two slopes again using AAP instead of MAP. Furthermore, although foliar *δ*^13^C values respond to aridity in a similar way for different C_3_ species and life forms (Prentice et al. 2011), the patterns might be different over times according to our finding.

Our results have valuable implications in methodology for future field sampling studies. Spatial variability of foliar *δ*^13^C in microhabitats can also affect its response patterns to precipitation at a large scale (Fig.3 and Table S1). Therefore, multiple sampling along the direction of the maximum environmental variance in each sampling site can reduce sampling error caused by microhabitat differences. Two transect studies in Inner Mongolia indicated that some species showed no linear regression in foliar *δ*^13^C values with aridity gradient (Su et al. 2000; Prentice et al. 2011). The reason for that result could be that the replication in each sampling site was low, or the replications did not represent the maximum environmental variance of the microhabitat surveyed. Alternatively, as grasslands had been increasingly grazed, the most common landscape is a scenario where grazed type and fenced type alternately occupy the temperate steppe of Inner Mongolia. This study also showed that there was lower foliar *δ*^13^C value in presence of grazed type than fenced type. It suggests that different land-use types (grazed or fenced) significantly affect foliar *δ*^13^C values of species. Hence, when we do field sampling along a transect in Inner Mongolia, all sampling sites are best occupied by visually homogeneous grassland disturbed by land-use types (grazed or fenced). Finally, we suggest for future investigations of the use of AAP in sampling year instead of MAP. It will yield more reliable results of the foliar *δ*^13^C in relation to precipitation along transect at a large geographical scales.

## Supporting Information

**Figure S1.** Two ecotypes of *Leymus chinensis* co-occurring in the field in Inner Mongolia. **Figure S2.** Difference in foliar *δ*^13^C of two ecotypes of *Leymus chinensis* in Inner Mongolia in 2011. **Figure S3.** One sampling sites in the Hulunbuir meadow steppe of Inner Mongolia. China.

**Table S1.** Results of the linear regression between foliar *δ*^13^C and actual annual precipitation using different samples size at each site along the transect of Inner.
