# Supplementary material for: Effects of sampling method on foliar δ13C of Leymus chinensis at different scales
Source: Ecol Evol. 2015 Feb 9;5(5):1068–75. doi: 10.1002/ece3.1401 (PMC4364821; doi:10.1002/ece3.1401)
Supplement: Supplementary file 1 [file ece30005-1068-sd1.docx]

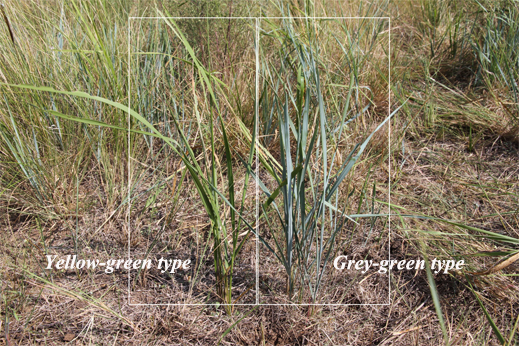


**Figure S1.** Two ecotypes of *Leymus chinensis* co-occurring in the field in Inner Mongolia. Left: the yellow-green type. Right: the grey-green type. Credit: Yanjie Liu. 2011.

**
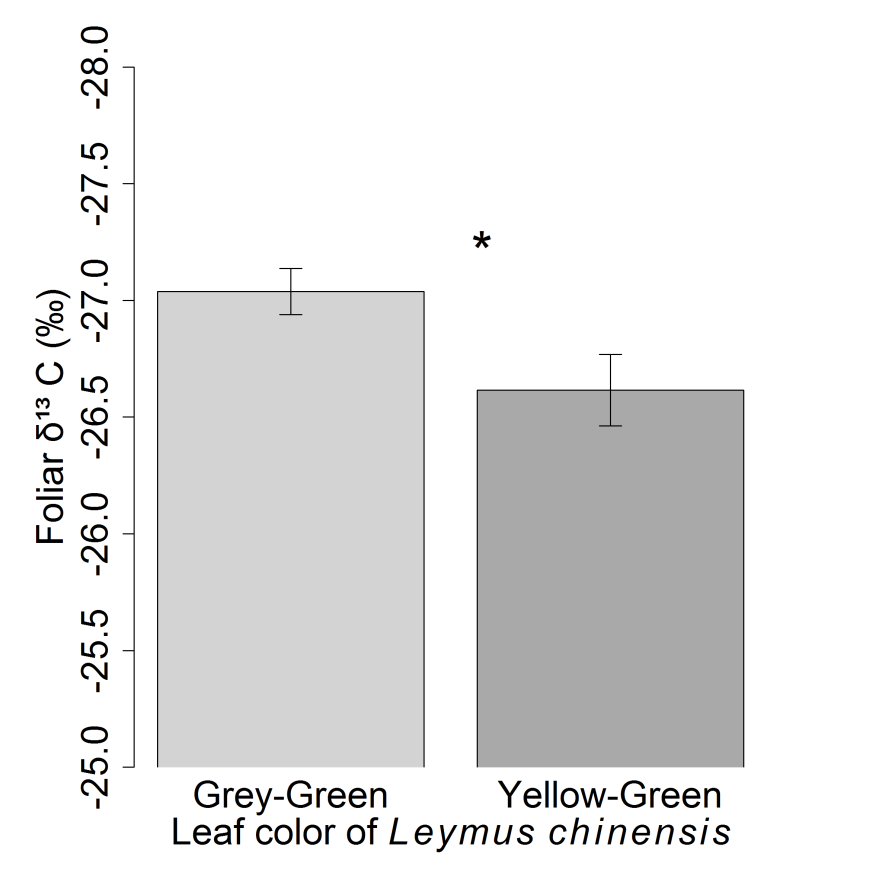
**

**Figure S2.** Difference in foliar δ^13^C of two ecotypes of *Leymus chinensis* in Inner Mongolia in 2011. Grey-Green and Yellow-Green refer to the leaf color for each ecotype. Error bars represent standard errors of the means. Level of significance: ‘*’ *p* < 0.05.

Sampling strategy: Four sites were selected in Inner Mongolia. China. in 2011 (1. 48°38.593' N. 116°48.935' E; 2. 48°27.197' N. 117°18.799' E; 3. 49°25.959' N. 118°48.209' E; 4. 44°28.759' N. 117°15.977' E). At each site. we first assessed the topography and identified a baseline along the hillside aspect on each site. Then. we collected five samples from five plots along the baseline. The distance between two adjacent plots along the baseline was 10 m. For each sample. we collected all mature leaves of 5–8 *L. chinensis* individuals randomly and mixed them. We tested the effect of site and ecotype on foliar δ^13^C using a two-way analysis of variance (ANOVA).


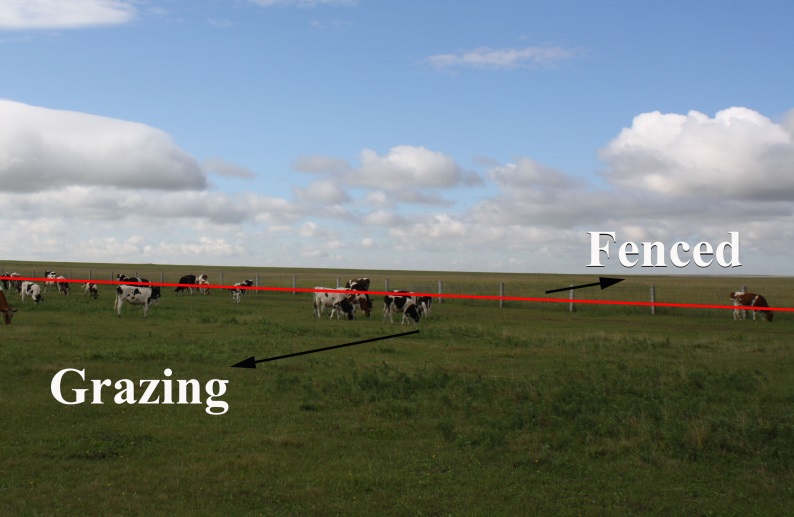


**Figure S3.** One sampling sites in the Hulunbuir meadow steppe of Inner Mongolia. China. The non-grazed area was fenced in 2006 and remained ungrazed since then. The grazed area was grazed by cattle animals from May to September. Credit: Yanjie Liu. 2010.
