# Supplementary material for: Effects of sampling method on foliar δ13C of Leymus chinensis at different scales
Source: Ecol Evol. 2015 Feb 9;5(5):1068–75. doi: 10.1002/ece3.1401 (PMC4364821; doi:10.1002/ece3.1401)
Supplement: Supplementary file 2 [file ece30005-1068-sd2.docx]

**Table S1.** Results of the linear regression between foliar δ^13^C and actual annual precipitation using different samples size at each site along the transect of Inner Mongolia from 2009 to 2011.

| *Year* | *samples* | *Intercept* | *Slope* | *R^2^* | *P-value* |
| --- | --- | --- | --- | --- | --- |
| 2009 | 1 | -23.0781 | -0.0084 | 0.5249 | **0.0117** |
| 2009 | 1 | -23.7422 | -0.0061 | 0.4234 | **0.0302** |
| 2009 | 1 | -23.9768 | -0.0052 | 0.2018 | 0.1657 |
| 2009 | 1 | -24.8205 | -0.0032 | 0.0834 | 0.3890 |
| 2009 | 1 | -23.3335 | -0.0086 | 0.4354 | **0.0272** |
| 2009 | 2 | -24.5013 | -0.0045 | 0.1758 | 0.0521 |
| 2009 | 2 | -24.2981 | -0.0051 | 0.1538 | 0.0711 |
| 2009 | 2 | -23.7402 | -0.0068 | 0.2613 | **0.0150** |
| 2009 | 2 | -24.6070 | -0.0040 | 0.1317 | 0.0970 |
| 2009 | 2 | -24.9835 | -0.0032 | 0.1016 | 0.1483 |
| 2009 | 3 | -24.5621 | -0.0045 | 0.1693 | **0.0174** |
| 2009 | 3 | -24.6505 | -0.0040 | 0.1264 | **0.0423** |
| 2009 | 3 | -24.1791 | -0.0052 | 0.2094 | **0.0074** |
| 2009 | 3 | -23.7414 | -0.0066 | 0.3033 | **0.0009** |
| 2009 | 3 | -24.2885 | -0.0049 | 0.2171 | **0.0063** |
| 2009 | 4 | -24.3672 | -0.0049 | 0.1748 | **0.0047** |
| 2009 | 4 | -24.2137 | -0.0051 | 0.2051 | **0.0020** |
| 2009 | 4 | -24.3869 | -0.0048 | 0.1706 | **0.0053** |
| 2009 | 4 | -24.3592 | -0.0047 | 0.1834 | **0.0037** |
| 2009 | 4 | -24.4181 | -0.0046 | 0.1718 | **0.0052** |
| 2009 | 5 | -24.4836 | -0.0046 | 0.1906 | **0.0009** |
| 2009 | 5 | -24.2535 | -0.0051 | 0.1908 | **0.0009** |
| 2009 | 5 | -24.3737 | -0.0048 | 0.1673 | **0.0019** |
| 2009 | 5 | -23.9602 | -0.0059 | 0.0932 | **0.0311** |
| 2009 | 5 | -24.0024 | -0.0059 | 0.0895 | **0.0349** |
| 2009 | All | -24.2120 | -0.0052 | 0.2008 | **0.0002** |
| 2010 | 1 | -24.3618 | -0.0035 | 0.0298 | 0.6334 |
| 2010 | 1 | -23.3516 | -0.0094 | 0.3712 | 0.0615 |
| 2010 | 1 | -24.0994 | -0.0061 | 0.1409 | 0.2851 |
| 2010 | 1 | -24.4179 | -0.0047 | 0.0699 | 0.4606 |
| 2010 | 1 | -24.6842 | -0.0037 | 0.0616 | 0.4894 |
| 2010 | 2 | -23.5794 | -0.0075 | 0.1515 | 0.0898 |
| 2010 | 2 | -24.2835 | -0.0057 | 0.1320 | 0.1154 |
| 2010 | 2 | -25.0608 | -0.0017 | 0.0074 | 0.7188 |
| 2010 | 2 | -24.4798 | -0.0046 | 0.0634 | 0.2840 |
| 2010 | 2 | -24.7108 | -0.0029 | 0.0218 | 0.5349 |
| 2010 | 3 | -23.3394 | -0.0081 | 0.1697 | **0.0237** |
| 2010 | 3 | -23.5735 | -0.0070 | 0.1412 | **0.0407** |
| 2010 | 3 | -23.9512 | -0.0061 | 0.1541 | **0.0319** |
| 2010 | 3 | -24.1519 | -0.0050 | 0.0660 | 0.1704 |
| 2010 | 3 | -24.3600 | -0.0047 | 0.0716 | 0.1529 |
| 2010 | 4 | -23.9338 | -0.0057 | 0.0829 | 0.0715 |
| 2010 | 4 | -23.7231 | -0.0069 | 0.1357 | **0.0193** |
| 2010 | 4 | -24.0483 | -0.0056 | 0.0803 | 0.0763 |
| 2010 | 4 | -24.3984 | -0.0047 | 0.0679 | 0.1044 |
| 2010 | 4 | -24.0652 | -0.0058 | 0.1031 | **0.0434** |
| 2010 | 5 | -23.9487 | -0.0060 | 0.1186 | **0.0143** |
| 2010 | 5 | -24.0380 | -0.0059 | 0.1075 | **0.0201** |
| 2010 | 5 | -24.2069 | -0.0052 | 0.0746 | 0.0549 |
| 2010 | 5 | -24.1947 | -0.0050 | 0.0798 | **0.0468** |
| 2010 | 5 | -24.2107 | -0.0051 | 0.0868 | **0.0378** |
| 2010 | All | -24.0101 | -0.0058 | 0.0948 | **0.0167** |
| 2011 | 1 | -23.6372 | -0.0088 | 0.2052 | 0.1617 |
| 2011 | 1 | -22.4686 | -0.0121 | 0.5402 | **0.0100** |
| 2011 | 1 | -23.4339 | -0.0099 | 0.3107 | 0.0597 |
| 2011 | 1 | -22.1217 | -0.0129 | 0.6660 | **0.0022** |
| 2011 | 1 | -22.8947 | -0.0115 | 0.4508 | **0.0237** |
| 2011 | 2 | -22.9136 | -0.0107 | 0.4404 | **0.0008** |
| 2011 | 2 | -22.7349 | -0.0119 | 0.4529 | **0.0006** |
| 2011 | 2 | -22.8301 | -0.0113 | 0.4315 | **0.0009** |
| 2011 | 2 | -22.5099 | -0.0124 | 0.4854 | **0.0003** |
| 2011 | 2 | -22.5634 | -0.0125 | 0.5118 | **0.0002** |
| 2011 | 3 | -22.8175 | -0.0114 | 0.4691 | **0.0000** |
| 2011 | 3 | -22.7240 | -0.0116 | 0.4611 | **0.0000** |
| 2011 | 3 | -22.8351 | -0.0113 | 0.4592 | **0.0000** |
| 2011 | 3 | -22.8683 | -0.0112 | 0.4584 | **0.0000** |
| 2011 | 3 | -22.7756 | -0.0118 | 0.4857 | **0.0000** |
| 2011 | 4 | -23.0117 | -0.0108 | 0.4254 | **0.0000** |
| 2011 | 4 | -22.8444 | -0.0113 | 0.4354 | **0.0000** |
| 2011 | 4 | -22.7528 | -0.0114 | 0.4556 | **0.0000** |
| 2011 | 4 | -22.8647 | -0.0111 | 0.4333 | **0.0000** |
| 2011 | 4 | -22.8231 | -0.0115 | 0.4800 | **0.0000** |
| 2011 | All | -22.8333 | -0.0113 | 0.4493 | **0.0000** |

Note：the number highlighted in bold in the table means significant regressions.
